# Effect of Inhalation of Aromatherapy Oil on Patients with Perennial Allergic Rhinitis: A Randomized Controlled Trial

**DOI:** 10.1155/2016/7896081

**Published:** 2016-03-13

**Authors:** Seo Yeon Choi, Kyungsook Park

**Affiliations:** Department of Nursing, College of Nursing, Chung-Ang University, 84 Heukseok-ro, Dongjak-gu, Seoul 156-756, Republic of Korea

## Abstract

This study aimed to investigate the effects of aromatherapy oil inhalation on symptoms, quality of life, sleep quality, and fatigue level among adults with perennial allergic rhinitis (PAR). Fifty-four men and women aged between 20 and 60 were randomized to inhale aromatherapy oil containing essential oil from sandalwood, geranium, and* Ravensara* or almond oil (the placebo) for 5 minutes twice daily for 7 days. PAR symptoms determined by Total Nasal Symptom Score (TNSS), the Rhinoconjunctivitis Quality of Life Questionnaire (RQLQ), sleep quality by Verran Synder-Halpern (VSH) scale, and fatigue level by Chalder Fatigue Scale (CFS) were assessed before and after intervention period. Compared with the placebo, the experimental group showed significant improvement in TNSS, especially in nasal obstruction. The aromatherapy group also showed significantly higher improvements in total score of RQLQ and CFS. These findings indicate that inhalation of certain aromatherapy oil helps relieve PAR symptoms, improve rhinitis-specific quality of life, and reduce fatigue in patients with PAR. In conclusion, inhalation of aromatherapy essential oil may have potential as an effective intervention to alleviate PAR.

## 1. Introduction

Perennial allergic rhinitis (PAR) is a season-independent chronic disorder induced by inflammation mediated by immunoglobulin E (IgE) after allergen exposure, with major symptoms including sneeze, rhinorrhea, and nasal obstruction [1]. It is one of the most frequent chronic diseases, occurring in approximately 500 million people, and causes various impairments including fatigue, cognitive dysfunction, depression, and degraded quality of life [2–5].

The most well-known mechanism of allergic rhinitis (AR) including PAR is antigen-antibody reaction, where allergen-specific sensitization results in mast cell degranulation and the release of inflammatory mediators [6]. Patients with such antigen-specific IgE antibodies present early phase symptoms including sneeze and rhinorrhea and late phase symptoms like nasal obstruction [7]. Though the antigen-antibody mechanism undoubtedly plays an important role in AR, it is not sufficient to explain hypersensitivity to specific chemical mediators and altered organ responsiveness in allergy patients. The recent studies suggested that autonomic nervous system (ANS) dysfunction, especially sympathetic hypofunction, are associated with hypersensitivity of the nasal mucosa in AR [8].

While PAR has diverse medical treatments including avoidance, immunotherapy, pharmacological treatment, and surgery, each of them has its limitations [7]. For example, antigen avoidance is not feasible for those who live in antigen-prone environments, while immunotherapy has adherence issue due to efficacy concerns among patients [1, 9, 10]. Pharmacological treatments including antihistamines and topical steroids may cause sedation and growth problem for children [11].

Despite warnings against their efficacy and side effects, the complementary and alternative medicines (CAM) have gained popularity [12, 13]. Aromatherapy, especially direct inhalation of aroma essential oil fragrance, has long been used for various inflammatory diseases [12]. Essential oils such as eucalyptus,* Ravensara*, and frankincense contain the monoterpenes like 1,8-cineol, alpha-terpineol, and alpha-pinene, which demonstrate anti-inflammatory and immunomodulatory effect [14–16]. Other essential oils like sandalwood, rich in santalol, are frequently used for relaxation or sedation, implying potential effect on a hypersensitive organ [17].

Despite its advantages and potentials, the aromatherapy has few scientific clinical trials on human PAR patients, especially directly measuring PAR symptoms and quality of life (QOL). The current study aims at exploring the effect of inhaling a mixture of aromatherapy oils on the symptoms and quality of life among PAR patients.

## 2. Materials and Methods

### 2.1. Study Design and Participants

A double-blinded, randomized controlled trial was designed to investigate the effects of inhalation of blended aromatherapy oil on subjective symptoms, quality of life, sleep quality, and fatigue in perennial allergic rhinitis patients. The detailed inclusion criteria included (1) age between 20 and 60 years, (2) written diagnosis of PAR by physician, (3) no experience of aromatherapy in the past, (4) no history of psychiatric illness, (5) no current medication or treatment for allergic rhinitis such as surgery and immunotherapy, (6) no disturbance of olfactory acuity, and (7) being free of allergies related to aromatherapy. Ninety men and women aged between 20 and 60 with perennial allergic rhinitis living in Seoul, South Korea, volunteered for the study between June and July 2015; of these, 21 did not meet the eligibility criteria and 7 withdrew their content to participate. The remaining 62 participants were told the purpose and protocol of the experiment and submitted written consent. To ensure all participants are PAR patients, all of the participants presented a written diagnosis from their physicians with a statement that the patients had perennial and chronic AR symptoms and that such diagnosis was based on tests like skin prick test. The study design and protocol were approved by the Ethical Review Committee of the Chung-Ang University (code 1041078-201504-HRBM-083-01), and all participants provided written informed consent.

### 2.2. Intervention

The subjects were assigned by a random number table into two groups; the experimental group inhaling the fragrance of the blended aromatherapy oil dissolved in almond carrier oil while the control group was inhaling pure almond carrier oil. For the experimental group, aromatherapy oils from three plants, including sandalwood, frankincense, and* Ravensara*, were blended and dissolved in almond oil at a concentration of 0.2% (v/v). For the control group, almond carrier oil was chosen as placebo due to its odorless and nonstimulant attributes [18, 19]. All the aromatherapy oils and almond oil were obtained from Neumond GmbH (Raisting, Germany).

For blinding purpose, only the compounder knew subject assignment, and neither the participants nor the investigators were aware of the allocation. Participants in the two groups received 14 bottles, each containing 1 mL of treatment oil; all bottles had the identical shape and color. Each participant was instructed to self-treat for 14 sessions, performed at 10 AM and 10 PM for 7 consecutive days. They were told to decant the contents of one bottle onto a fragrance pad, sit in a stable and comfortable place, position the pad 30 cm away from their nose, and inhale the fragrance for five minutes with normal breathing [20].

### 2.3. Outcome Measurements and Data Collection

Pretrial assessments were performed on the day before day 1 of the 7-day intervention, while posttrial assessments were performed on day 8. The pretrial surveys included general characteristics, subjective symptoms by Total Nasal Symptom Score (TNSS), self-assessed qualify of life by Rhinoconjunctivitis Quality of Life Questionnaire (RQLQ), sleep quality measured by Verran Synder-Halpern (VSH) scale, and fatigue by Chalder Fatigue Scale (CFS). Posttrial surveys included all of the above parameters, except for general characteristics. Patients were asked to recall the four previous weeks for baseline measurement during the 7 days of treatment period for posttest measurement. To minimize the effect of data collection time, all surveys and measurements were conducted between 9 AM and 10 AM [20].

#### 2.3.1. PAR Symptoms

As one of the two primary outcome variables for aromatherapy efficacy, mean change in Total Nasal Symptom Score (TNSS) was measured between the baseline and day 8. The current study used the four components of sneeze, rhinorrhea, itchy nose, and nasal obstruction. Each was scored on a 4-point scale from 0 to 3 (0 = none, 1 = mild, 2 = moderate, and 3 = severe) giving TNSS range from 0 to 12 [21].

#### 2.3.2. Disease-Specific Quality of Life (QoL)

The other primary outcome measure was the improvement in patients' quality of life, assessed by mean change in Rhinoconjunctivitis Quality of Life Questionnaire (RQLQ) score. RQLQ consists of 28 questions on a 7-point scale (0 = not impaired at all, 6 = severely impaired) in 7 domains: activities limitation, sleep problems, nose symptoms, eye symptoms, non-nose/eye symptoms, practical problems, and emotional function [22]. Total score and seven domain scores were compared.

#### 2.3.3. Sleep Quality

As a secondary outcome measure, this study measured mean change in sleep quality using Verran Synder-Halpern (VSH) scale. VSH consists of eight visual analog scales (VAS) to capture characteristics of sleep latency, fragmentation, length, and depth. Each item is set to a 0–100 response scale. A total score, representative of overall sleep quality, can be calculated by summating each item response [23].

#### 2.3.4. Fatigue

The other secondary outcome measure is mean change in fatigue using Chalder Fatigue Scale (CFS). The CFS is a 14-item questionnaire, each item being scored from 0 to 3, generating a score between 0 and 42 [24].

### 2.4. Statistical Analysis

All data are presented as mean ± standard deviation, with all statistical analyses were performed using SPSS version 20.0 (SPSS Inc., Chicago, IL, USA). Intergroup comparisons of any normally distributed variable were performed using Student's two-sample *t*-test. Nonnormally distributed variables were compared using the Mann-Whitney *U* test. Within group, comparisons of normally distributed and non-normally distributed variables were assessed using paired *t*-tests and Wilcoxon signed rank tests, respectively. A *P* value < 0.05 was defined as statistically significant.

## 3. Results

### 3.1. General Characteristics of the Participants and Test of Homogeneity

**Figure 1:**
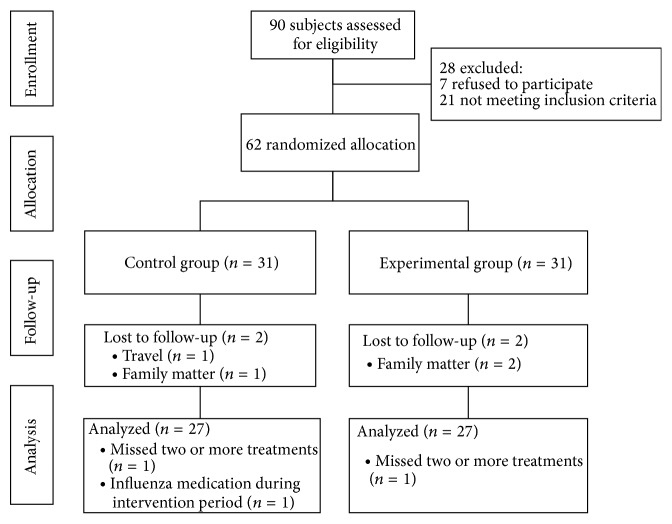
Study flow diagram.

From a total of 90 volunteers, 21 did not meet the eligibility criteria and 7 withdrew their content to participate. The remaining 62 patients were randomized and evenly assigned to the control group (*n* = 31) and the experimental group (*n* = 31). Two were lost to follow-up due to travel and family matter from the control group while two were lost to follow-up due to family matter from the experimental group. In addition, four patients were excluded from data analysis due to serious protocol violations: one from the control group and two from the experimental group due to missing two or more treatments and one from the control group due to influenza medication during the intervention [20]. Thus, data were collected and analyzed for 54 men and women, including 27 who received almond oil and 27 who received blended aromatherapy oil (Figure 1). There were no significant differences among the two groups in general characteristics and baseline outcome measures, indicating statistical homogeneity (Table 1).

### 3.2. Effect of Aromatherapy Oil on PAR Symptoms

After the 7 days of intervention, TNSS in the aromatherapy group reduced significantly more than the almond oil group (*P* = 0.022) while both groups reduced in total score: the aromatherapy group from 6.815 ± 2.202 to 3.259 ± 1.403 and the almond oil group from 6.444 ± 2.532 to 4.593 ± 2.485.

Among the four symptoms, the two groups showed significant difference in the mean change of “nose obstruction” (*P* = 0.035), while “sneeze,” “runny nose,” and “itchy nose” demonstrated a tendency of higher improvement among the aromatherapy group (Table 2).

### 3.3. Effect of Aromatherapy Oil on RQLQ

After the intervention, the overall RQLQ score in the experiment group decreased significantly more than the placebo group (*P* = 0.002). Like TNSS, the two groups reduced in the overall RQLQ score: the aromatherapy group from 1.870 ± 0.561 to 0.714 ± 0.436 and the almond oil group from 1.903 ± 0.614 to 1.315 ± 0.770. Out of the seven domains in RQLQ assessment, five domains including practical problem, sleep, nose, activeness, and emotion domains showed significant differences between the two groups. The “eye” and “non-nose/eye physical” domains did not show significant difference though the experimental group had strong tendencies of higher improvement than the control group (Table 3).

### 3.4. Effect of Aromatherapy Oil on Sleep Quality and Fatigue

**Figure 2:**
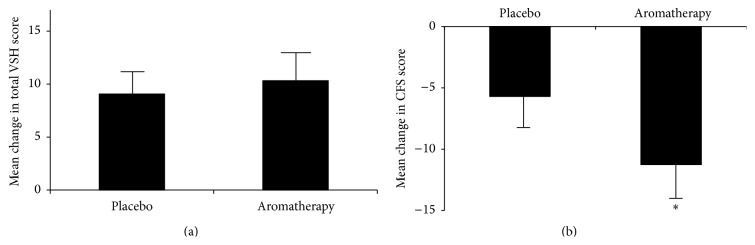
Effect of aromatherapy oil on (a) the Verran Synder-Halpern (VSH) sleep quality scale and (b) Chalder Fatigue Scale (CFS). Values are expressed as mean ± SEM. ^*∗*^ *P* < 0.05.

The VSH sleep quality score increased in both groups, and there was no significant difference in mean changes between the two groups. In Chalder fatigue score, the experiment group improved significantly more than the placebo group (*P* = 0.021) while both groups reduced in total score: the aromatherapy group from 35.000 ± 7.000 to 23.741 ± 4.703 and the almond oil group from 33.481 ± 7.678 to 27.778 ± 5.938 (Figure 2).

## 4. Discussion

The current study was designed to investigate the effects of aromatherapy oil inhalation on subjective perception of PAR symptoms, quality of life, sleep quality, and fatigue among PAR patients. Fifty-four men and women aged between 20 and 60 years inhaled blended aromatherapy oil or almond oil twice a day for seven days, and the effects on TNSS, RQLQ, VSH, and CFS were measured. Compared with the placebo, the experimental group showed significant improvement in TNSS, RQLQ, and CFS.

TNSS and the “nose” domain of RQLQ directly measure the nasal symptoms of PAR patients. The significant difference in mean change of the two variables indicates that the inhalation of the blended oils helps alleviate PAR symptoms. The three essential oils used for the intervention contain a number of chemical compositions with anti-inflammatory and anti-allergic effect. For example, 1,8-cineole, which is abundant in* Ravensara*, has proven its anti-inflammatory effect by decreasing the production of inflammatory mediators [14, 25, 26]. Alpha-terpineol, another major component of* Ravensara*, also proved its anti-inflammatory effect [27]. Alpha-pinene in Frankincense oil reduces allergic symptoms and inflammatory mediator like interleukin-4 (IL-4) among mice with AR [28].

Allergic reaction of AR patients develops in two different patterns along time sequence: the early reaction represented by sneeze and rhinorrhea and the late reaction represented by nasal obstruction [7]. While the early reaction involves stimulated mast cells secreting chemical mediators such as histamines, the late reaction is mainly caused by eosinophil chemotaxis where inflammatory cells migrate to nasal mucosa and remodel normal nasal tissue [7]. From this antigen-antibody reaction perspective, there are two pathways of allergic symptom alleviation: the blended aromatherapy oil reduces the proinflammatory mediators, or it even reduces the number of inflammatory cells.

Looking into the TNSS result, “nasal obstruction” was the only symptom which showed a significant improvement by the aromatherapy oil inhalation. A previous study on patients with allergic rhinopathy showed that a nasal spray containing* Ravensara* essential oil reduced the eosinophils granulocytes and mast cells without eosinophil and metachromatic granules over the intervention period, implying potential for not only soothing AR symptoms but also preventing the source of them [15]. Another study on mice with AR proved that alpha-pinene actually suppressed migration of eosinophils and mast cells [28]. In summary, the blended aromatherapy oil may not only inhibit inflammatory mediator but also recover the sensitized cells.

The effect of the aromatherapy oil on PAR symptoms can be also explained through its effect on autonomic nervous system (ANS) imbalance. Santalol in sandalwood essential oil is associated with elevating parasympathetic nervous system (PNS) for relaxing and sedative effect, and it seems that santalol stimulates parasympathetic nerves in the hypersensitive nasal mucosa for less allergic reactions [29].

Besides the nasal symptoms, the aromatherapy group demonstrated significantly higher improvement in five domains of RQLQ except for “eye” and “non-nose/eye physical” domains. Sandalwood essential oil is known for its “harmonizing” effect, where it relaxes in terms of physiology while it stimulates in terms of behavior [29]. This effect is also demonstrated in the behavioral domains of RQLQ including “activity limitation,” “practical problems,” and “emotional function.”

After the 7-day intervention, the aromatherapy group demonstrated significantly higher improvement in fatigue measured by CFS. In addition, the aromatherapy group improved significantly more than the placebo in the sleep domain of RQLQ and a similar tendency in the VSH score. As PAR symptoms including nasal obstruction cause sleep disorder and fatigue, it can be inferred that alleviated PAR symptoms among the control group help improve sleep quality and reduce their fatigue level [1, 2]. These results are aligned with the previous findings that improvement in AR symptoms would lead to improvement in sleep quality and chronic fatigue is frequently accompanied with AR symptoms [1, 24]. In addition, a number of previous studies have proved that aromatherapy essential oils including lavender, rosemary, and sandalwood help improve sleep quality and decrease fatigue [30].

Comparing the sleep domain in RQLQ survey and the VSH survey, we can find that VSH is inclusive of RQLQ: the questions on time to sleep, sleep fragmentation, and sleep depth are common, while VSH survey adds sleep duration and overall sleep quality. In the current study, the two groups showed significant difference in sleep fragmentation and sleep depth in both questionnaires. In contrast, the two groups were not significantly different in sleep duration, time to sleep, and overall sleep quality. Therefore, the result on sleep quality is generally consistent in both surveys and the apparently conflicting results are mainly due to the difference in survey items.

Reviewing the intragroup comparison across the outcome measures, the control group showed noticeable improvements. This indicates that the placebo effect was not ignorable. Every participant had 14 5-minute inhalation sessions over 7 days, with some lifestyle control like alcohol consumption and smoking. This requires time keeping, stable breathing and inhalation, and self-attentiveness. Stable breathing and inhalation in particular may have relaxation and sedative effect, leading to higher sleep quality [31]. In addition, we can expect some “Hawthorne effects,” where the control group shows sizeable improvement without any intervention [32]. In addition, there were some factors which differed between the pretrial environment and the posttrial environment, such as weather, temperature, and humidity. They might affect the intragroup comparison, but not intergroup one.

Most of the previous studies are either animal trials or humans with non-AR diseases, including asthma and bronchitis. Though Remberg's trial involved AR patients treated with 1,8-cineole, it showed only an immediate effect [25]. And most of those studies were conducted in highly controlled environment. In contrast, the current study reports that the inhalation of the aromatherapy oil has a potential for improving AR in human adults over a certain period of time. In addition, these results were obtained in less controlled environment where the participants maintained their normal lifestyle, indicating the practical value of aromatherapy.

The current study has its limitations including (1) no follow-up measurement after posttreatment to investigate the duration of efficacy, (2) no biomarkers for PAR symptoms such as IgE, and (3) aromatherapy efficacy depending on the concentration level. These limitations should be considered in the future studies.

## 5. Conclusion

In conclusion, the current randomized controlled trial showed that the inhalation of blended oil from* Ravensara*, frankincense, and sandalwood alleviated subjective symptoms, improved the disease-specific quality of life, and reduced fatigue among adult patients with PAR. This intervention also has potential for improvement in sleep quality.

These findings indicate that aromatherapy oil inhalation can be used as a safe and effective complementary intervention to reduce PAR symptoms and improve quality of life among the patients.

## Figures and Tables

**Table 1:** Homogeneity test for general characteristics and measurement variables.

Characteristics or variables	Placebo (*N* = 27)	Aromatherapy (*N* = 27)	Total (*N* = 54)	*P* value
Age (year)	30.852 ± 11.302	28.889 ± 9.010	29.870 ± 10.172	0.652^*∗*^
TNSS (score)				
Overall	6.444 ± 2.532	6.815 ± 2.202	6.630 ± 2.358	0.569
Sneeze	1.481 ± 0.753	1.519 ± 0.580	1.500 ± 0.666	0.885^a^
Runny nose	1.852 ± 0.864	1.815 ± 0.736	1.833 ± 0.795	0.825^a^
Itchy nose	1.630 ± 0.792	1.630 ± 0.839	1.630 ± 0.808	0.244^a^
Nose obstruction	1.556 ± 0.801	1.852 ± 0.818	1.704 ± 0.816	0.969^a^
RQLQ (score)				
Overall	1.903 ± 0.614	1.870 ± 0.561	1.887 ± 0.583	0.837
Activity limitation	2.605 ± 0.925	2.568 ± 0.772	2.586 ± 0.844	0.924
Sleep problems	1.778 ± 1.013	1.728 ± 1.004	1.753 ± 0.999	0.972^a^
Nose symptoms	2.630 ± 0.861	2.639 ± 0.606	2.634 ± 0.738	0.957
Eye symptoms	1.852 ± 1.057	1.574 ± 0.914	1.713 ± 0.989	0.310
Non nose/eye symptoms	2.185 ± 0.814	1.984 ± 0.743	2.085 ± 0.778	0.390
Practical problems	1.062 ± 0.722	1.358 ± 0.852	1.210 ± 0.796	0.180
Emotional function	0.935 ± 0.903	1.167 ± 0.893	1.051 ± 0.897	0.386
VSH scale (score)	38.570 ± 11.598	42.159 ± 10.025	40.365 ± 10.889	0.229
CFS (score)	33.481 ± 7.678	35.000 ± 7.000	34.241 ± 7.317	0.451

TNSS, total nasal symptoms score; RQLQ, Rhinoconjunctivitis quality of life questionnaire; VSH, Verran Synder-Halpern; CFS, Chalder fatigue scale.

Data reported as mean ± standard deviation.

Student's 2-sample *t*-test. ^a^Mann-Whitney *U* test.

**Table 2:** Effect of aromatherapy oil on allergic rhinitis symptoms (*N* = 54).

Characteristics or variables	Placebo (*N* = 27)	Aromatherapy (*N* = 27)	*P* value
TNSS (score)			
Overall	−1.852 ± 2.125	−3.556 ± 2.486	0.022^a^
Sneeze	−0.370 ± 0.742	−0.630 ± 0.742	0.179^a^
Runny nose	−0.630 ± 0.926	−1.000 ± 0.832	0.219^a^
Itchy nose	−0.556 ± 1.013	−1.074 ± 0.829	0.063^a^
Nose obstruction	−0.370 ± 0.742	−0.852 ± 0.864	0.035^a^

TNSS, total nasal symptoms score.

Data reported as mean ± standard deviation.

Student's 2-sample *t*-test. ^a^Mann-Whitney *U* test.

**Table 3:** Effect of aromatherapy oil on allergy rhinitis-specific quality of life (*N* = 54).

Characteristics or variables	Placebo (*N* = 27)	Aromatherapy (*N* = 27)	*P* value
RQLQ (score)			
Overall	−0.589 ± 0.669	−1.156 ± 0.579	0.002
Activity limitation	−0.589 ± 0.948	−1.281 ± 0.909	0.008
Sleep problems	−0.574 ± 0.865	−1.185 ± 1.000	0.021
Nose symptoms	−0.833 ± 0.805	−1.622 ± 0.850	0.001^a^
Eye symptoms	−0.659 ± 0.925	−1.122 ± 0.771	0.051
Non nose/eye symptoms	−0.722 ± 0.827	−1.119 ± 0.792	0.078
Practical problems	−0.407 ± 0.948	−0.974 ± 0.778	0.020
Emotional function	−0.241 ± 0.944	−0.856 ± 0.943	0.031^a^

RQLQ, Rhinoconjunctivitis quality of life questionnaire.

Data reported as mean ± standard deviation.

Student's 2-sample *t*-test. ^a^Mann-Whitney *U* test.
