# Corrigendum to “Effect of Inhalation of Aromatherapy Oil on Patients with Perennial Allergic Rhinitis: A Randomized Controlled Trial”

**DOI:** 10.1155/2016/2103616

**Published:** 2016-10-11

**Authors:** Seo Yeon Choi, Kyungsook Park

**Affiliations:** Department of Nursing, College of Nursing, Chung-Ang University, 84 Heukseok-ro, Dongjak-gu, Seoul 156-756, Republic of Korea

In the article titled “Effect of Inhalation of Aromatherapy Oil on Patients with Perennial Allergic Rhinitis: A Randomized Controlled Trial,” [1] there were some errors that should be corrected as follows.

In the abstract section “geranium” should be changed to “frankincense.” Also, the Latin botanical names of the three essential oils should have been presented as Santalum album (Sandalwood), Ravensara aromatica (Ravensara), and Boswellia carterii (Frankincense).

The authors confirm using “Ravensara” essential oil, rather than the similarly named “ravintsara.”
